# Upfront triple combination therapy with selexipag: insights from a real world cohort in Chinese patients with pulmonary arterial hypertension

**DOI:** 10.3389/fcvm.2026.1745171

**Published:** 2026-05-21

**Authors:** Xiaopei Cui, Xiaoyu Hu, Xiaoteng Qin, Haijun Li, Xia Xu, Hui Liu, Qiushang Ji

**Affiliations:** 1 Department of Geriatric Medicine & Laboratory of Gerontology and Anti-Aging Research, Qilu Hospital of Shandong University, Jinan, Shandong Province, China; 2 Department of Cardiology, Qilu Hospital, Cheeloo College of Medicine, Shandong University, Jinan, Shandong, China; 3 Department of General Practice, Qilu Hospital, Cheeloo College of Medicine, Shandong University, Jinan, Shandong, China

**Keywords:** pulmonary arterial hypertension, right heart, risk assessment, selexipag, survival, triple combination

## Abstract

**Aim:**

Selexipag has demonstrated safety and efficacy in the treatment of pulmonary arterial hypertension (PAH). However, the strategy of triple therapy involving selexipag and its impact on right heart remodeling requires further clarification in Chinese patients with PAH.

**Methods and results:**

This investigation was conducted as a single-center, retrospective study. Patients with PAH who presented to Qilu Hospital of Shandong University from February 2022 to November 2023 with a definitive diagnosis of Group 1 PAH and who received a triple targeted therapy based on selexipag were included for a safety and efficacy evaluation. Data for the French pulmonary hypertension network non-invasive risk assessment, echocardiogram parameters, and clinical data were collected. Comparisons between initial and early sequential triple combination were conducted. A total of 153 patients were entered into the safety analysis and 146 patients were included in the efficacy analysis. No unknown side effects were reported. After selexipag treatment for 29 weeks in median, 96 (65.8%) reached WHO FC II compared to 39 (26.7%) at baseline (*p* < 0.001). The 6MWD increased from 414 ± 108 m to 480 (420, 506) m (*p* < 0.001), the NT-proBNP level decreased from 928 (307, 1,923) pg/mL to 455 (134, 1,678) pg/mL (*p* < 0.001). The number of patients that had 3, 2, 1 or 0 low-risk criteria at baseline were 18 (12.3%), 26 (17.8%), 32 (21.9%), and 70 patients (47.9%), respectively. The risk stratification of patients improved significantly during follow-up, with the number of patients meeting 3 low-risk criteria increasing to 74 (50.7%). There was significant improvement in right atrial area, right ventricle diameter, and tricuspid annular plane systolic excursion. The average follow-up period was 33 ± 10 months. The hazard ratio for the risk of experiencing a first disease progression event was 0.177 (*p* = 0.036, 95% CI: 0.035–0.898) for initial triple combination therapy, compared to early sequential selexipag add-on therapy.

**Conclusion:**

Triple-targeted drug therapy containing selexipag can be safely and effectively used in Chinese PAH patients, significantly enhancing exercise capacity, improving right heart structure and function, and optimizing risk stratification. Selexipag-based initial triple therapy may offer demonstrable benefits for long term prognosis.

## Introduction

Pulmonary arterial hypertension (PAH) is a progressive devastating disease resulting in right heart failure and ultimately death mostly with a higher prevalence in female patients (1). During the past decade, the survival rate is much improved with PAH combination targeted drug therapy (2). According to the AMBITION study, initial dual combination with endothelial receptor antagonists (ERAs) and phosphodiesterase type 5 inhibitors (PDE5is) decreased the rate of clinical worsening in PAH patients, and initial dual combination therapy was recommended by both 2015 and 2022 ESC/ERS Guidelines for the diagnosis and treatment of pulmonary hypertension (3, 4). However, approximately 50% of patients treated initially with the combination therapy of ambrisentan and tadalafil for 2 years remain in a medium/high risk status (5). Data from both the French and Canadian PAH registries show that initial dual therapy with an ERA and a PDE5 inhibitor confers no survival advantage over initial monotherapy beyond five years (6, 7). These findings suggest that adding a prostacyclin-pathway agent such as selexipag to upfront combination regimens may be necessary to meaningfully improve long-term prognosis in PAH. However, the optimal timing of such intervention remains uncertain.

Selexipag is an oral selective IP receptor agonist for PAH treatment (8). There is cumulative evidence on the efficacy and safety of selexipag sequential combination in PAH treatment (9, 10). Recently, the proper timing for selexipag add-on therapy captured wide attention. Both *post-hoc* analysis from GRIPHON study and data from US Komodo claims database suggested that early addition of oral selexipag to double oral therapy with an ERA plus a PDE5i within 6 months after PAH diagnosis is associated with a lower risk of hospitalization and disease progression in patients with PAH (11).

There is growing evidence that PAH prognosis is strongly associated with right heart structure and function (12–14). Previously, we published the first selexipag based triple combination cohort study suggested that selexipag is effective in treating Chinese PAH patients in a small exploratory cohort, resulting in improved risk status. However, the reduction in right atrial area did not reach a significant change, which is a critical prognostic parameter for PAH patients (9, 14). In this study, in which validated cohorts were established, we further focused on the timing of initiation of selexipag based triple combination therapy and its effect on right heart remodeling in PAH patients as well as timing to the onset of a clinical worsening event.

## Methods

### Study design and patient selection

This is a single-center, retrospective study registered on the Chinese Clinical Trial Registry: identifier ChiCTR2300067393. Patients with pulmonary arterial hypertension who presented to Qilu Hospital of Shandong University from February 2022 to November 2023 were screened. The inclusion criteria were: (1) ≥18 years old; (2) definitive diagnosis of Group 1 PAH by right heart catheterization (RHC); (3) patients with idiopathic PAH (IPAH), heritable PAH (HPAH), post-operative PAH associated with congenital heart disease (postoperative CHD-PAH), or PAH associated with connective tissue diseases (CTD-PAH); (4) receiving selexipag-based triple oral therapy; (5) Patients with newly diagnosed WHO Group 1 PAH who had received no prior PAH-specific therapy, or those with established WHO Group 1 PAH stably treated with dual ERA/PDE5 inhibitor therapy for ≥3 months. Exclusion criteria were: (1) patients with PAH related to uncorrected congenital heart disease (having treat-to-repair therapy); (2) patients with severe obstructive pulmonary disease, or (3) patients with primary malignant tumors. Patients who initiated selexipag-based triple oral therapy within 1 month following a diagnosis of PAH were categorized as the initial set. Those who received selexipag as an add-on to stable ERA and PDE5i dual combination therapy were classified as the sequential set; within this group, patients who commenced selexipag add-on therapy within 12 months post-PAH diagnosis were identified as the early sequential set. Additionally, patients who switched to selexipag from treprostinil were designated as the transition set.

The cutoff date for risk stratification follow-up data collection was April 30, 2024. For the analysis, the safety set included all patients taking at least one dose of selexipag, while the efficacy set included patients taking continuous selexipag treatment over 12 weeks and undergoing at least one risk assessment during follow-up. The study was approved by the Ethics Committee on Scientific Research of Shandong University Qilu Hospital (reference number: KYLL-202206-031) and written informed consent was obtained.

Selexipag dose titration, and the process for transition from treprostinil to selexipag was the same as we previously reported (9). The primary study endpoint was improvement in the number of low-risk indices on the French noninvasive risk assessment at the first follow-up after initiation of treatment with selexipag. Low-risk indices include: cardiac functional class (WHO) Ⅰ/Ⅱ, six-minute walking distance（6MWD）> 440 m, and NT-proBNP < 300 pg/mL. The improvement in the French non-invasive risk assessment was defined by an increase in the number of low-risk indicators met. The secondary endpoints were (1) right heart remodeling reversion, defined as normalization of all 3 parameters measured by echocardiogram, including right atrial area (RAA) < 18 cm^2^, RV < 28 mm and tricuspid annular plane systolic excursion (TAPSE) ≥ 18 mm, and (2) side effects of selexipag leading to medication withdraw. The WHO FC was determined by an experienced physician, and the same technician performed transthoracic echocardiography during follow-up visits. The echocardiogram was conducted by the same echocardiography specialist. The right ventricular dimension was measured as the anteroposterior diameter. In the parasternal long-axis view, the sampling line was oriented perpendicular to the left ventricular posterior wall and interventricular septum, passing through the mitral valve tips and extending to the inner wall of the right ventricle. The distance between the right ventricular side of the interventricular septum and the inner wall of the right ventricle was measured, with a value less than 28 mm considered normal. The right atrial area also serves as an indicator of right ventricular diastolic function. The apical RA-focused view was employed. The sampling line was traced from the inner aspect of the tricuspid annulus, along the right atrial side of the interatrial septum, the right atrial roof, the right atrial lateral wall, and back to the lateral aspect of the tricuspid annulus, connecting to the starting point. The area enclosed by this trace was defined as the right atrial area, with a normal value being less than 18 cm^2^. TAPSE was chosen as the indicator of right ventricular systolic function due to its robust evidence base, simplicity of operation, and good reproducibility. The apical RV-focused view was utilized. The sampling line was aligned perpendicular to the lateral aspect of the tricuspid annulus. Using M-mode ultrasound, the vertical distance from diastole to systole was measured to obtain TAPSE, with a value less than 18 mm considered abnormal. Events including hospitalization, death, and initiation of parenteral PGIs associated with PAH progression were observed until May 31, 2025 or until the withdrawal date for patients who discontinued selexipag. For patients with multiple events, only the first event was noted.

### Statistical analyses

Statistical analyses were conducted using SPSS Statistics (Version 26.0) and GraphPad Prism (Version 10). Categorical data are presented as counts or percentages. Normal distribution was evaluated using the Kolmogorov–Smirnov test. Continuous variables are presented as the mean ± standard deviation when distributed normally, or otherwise as the median with the interquartile range (Q1, Q3). Paired t-test, paired rank sum test or the chi-square test were used to compare the differences between baseline and follow-up values where appropriate. Survival outcomes were estimated using the Kaplan–Meier method. The proportional hazards assumption was formally assessed using scaled Schoenfeld residuals. Significant differences were defined as *p* < 0.05 (two-tailed test).

## Results

### Baseline characteristics of the cohort

**Figure 1:**
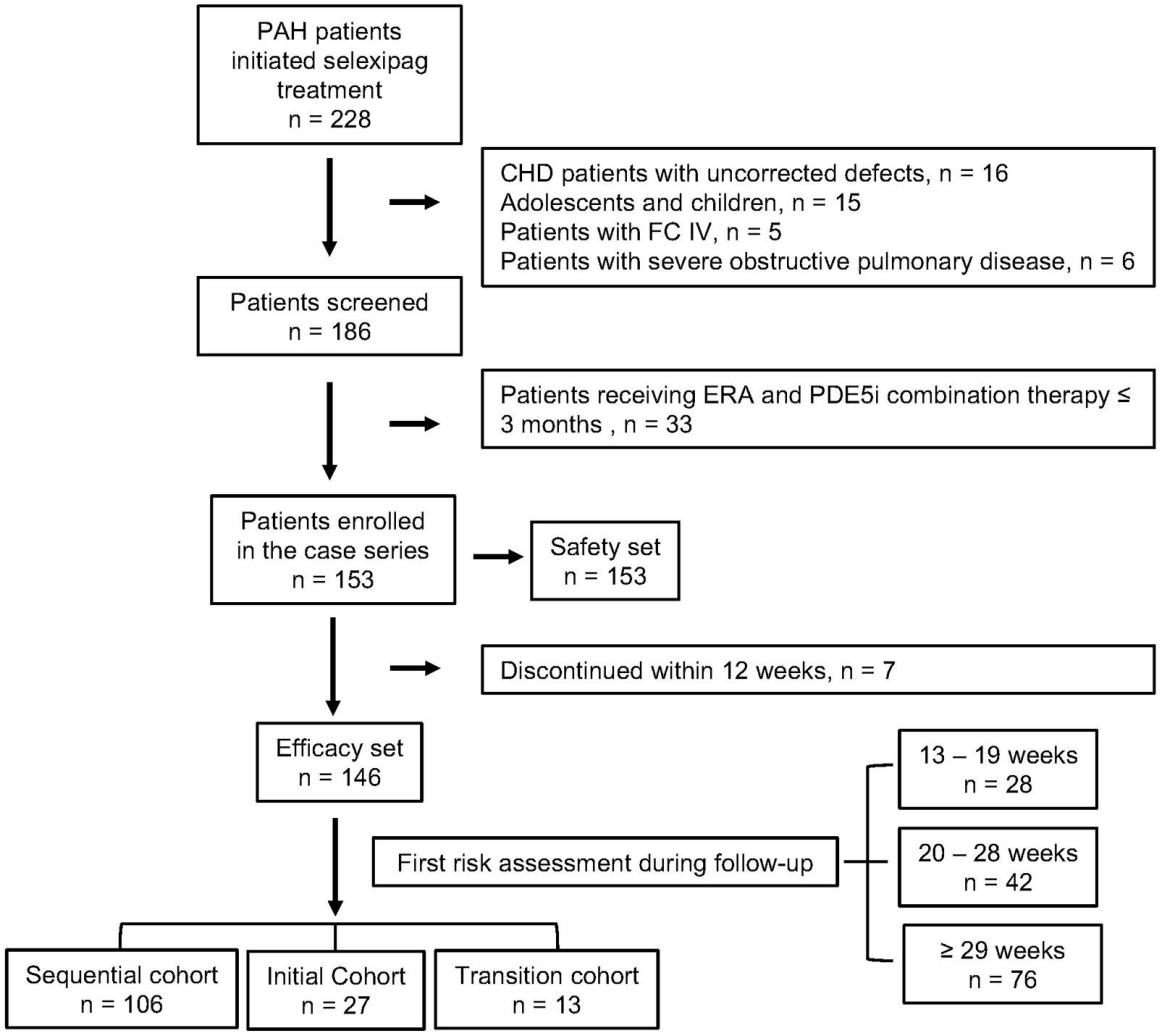
Flow chart of patients’ selection.

A total of 228 patients initiated treatment with selexipag, and 42 cases were excluded, including 16 patients with unrepaired CHD-PAH, 15 who were <18 years old, 5 who were in WHO functional class IV, and 6 who had combined moderate-to-severe obstructive ventilatory dysfunction. In addition, further exclusion of 33 patients who had been treated with the combination of ERA and PDE5i for more than 1 month and less than 3 months, a total of 153 patients were entered into the safety cohort analysis, of which 7 patients discontinued the use of selexipag within 12 weeks of treatment. Thus, 146 patients treated for ≥12 weeks were included in the outcome analyses. The median duration until the first risk stratification was 29 weeks, with an interquartile range of 21 to 36 weeks. Specifically, 28 patients completed their first risk stratification between week 13 and week 19, 42 patients between week 20 and week 28, and 77 patients at week 29 or later (Figure 1). The average follow-up period for clinical event was 33 ± 10 months.

### Changes of risk assessment of the efficacy cohort

**Figure 2:**
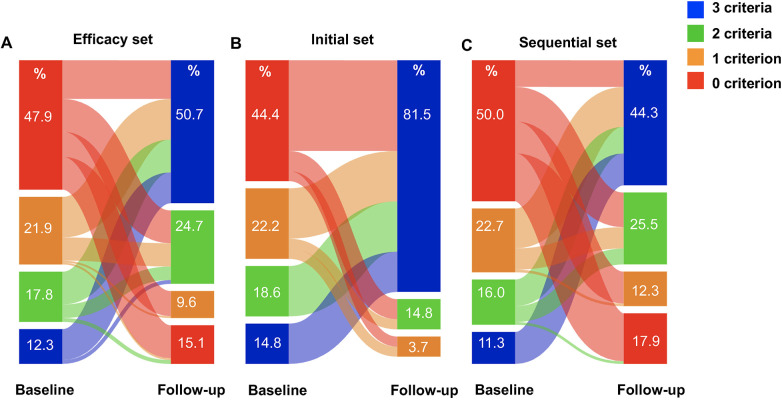
Change of risk assessment at baseline and follow-up. Change in **(A)** whole efficacy set (*n* = 146); **(B)** initial set (*n* = 27) and **(C)** sequential set (*n* = 106).

In the efficacy set, 120 (82.2%) patients were female, with a mean age of 31.9 years and a median time from definitive diagnosis to initiation of selexipag of 36 months. In terms of etiologic distribution, 90 (61.6%) patients had IPAH/HPAH, 29 (19.9%) patients had CHD-PAH after defect repair, and 27 (18.5%) patients had CTD-PAH. As shown in Table 1, 27 newly diagnosed patients received initial triple therapy that included selexipag (initial set), 106 patients had sequential triple therapy (sequential set), and 13 patients transitioned from treprostinil to selexipag (transition set). There were 20 patients who had sequential selexipag add-on to upfront ERA and PDE5i dual combination within 12 months after PAH diagnosis, defined as the early sequential group. As shown in Table 2, before selexipag initiation, 39 (26.7%) patients were at WHO FC II and the other 107 (73.3%) patients were at WHO FC III. After selexipag treatment for 29 weeks in median, 96 (65.8%) reached WHO FC II (*p* < 0.001). The 6MWD increased from 414 ± 108 m to 480 (420, 506) m (*p* < 0.001), the NT-proBNP level decreased from 928 (307, 1,923) pg/mL to 455 (134, 1,678) pg/mL (*p* < 0.001). Eighteen (12.3%) patients met 3 low-risk criteria at baseline, the number of patients meeting 2 low-risk criteria was 26 (17.8%), the number of patients meeting 1 low-risk criterion was 32 (21.9%), and 70 patients (47.9%) met 0 low-risk criteria. The risk stratification of patients improved significantly during 29 weeks median follow-up, with the number of patients meeting 3 low-risk criteria increasing to 74 (50.7%), the number of patients meeting 2 low-risk criteria being 36 (24.7%), the number of patients meeting 1 low-risk criterion being 14 (9.6%), and the number of patients who did not meet any low-risk criteria decreased to 22 (15.1%) (Figure 2A). Regardless of the time to the first risk stratification, the number for low-risk criteria increased significantly, as demonstrated in Supplementary Tables S1–S3. The transition set comprised 13 patients, among whom exercise capacity and right heart structure remained stable during follow-up, while NT-proBNP levels exhibited a continuous decline, as summarized in Supplementary Table S4.

**Table 1:** Baseline characteristics.

Variable	Efficacy set *n* = 146	Initial set *n* = 27	Sequential set *n* = 106	Early sequential set *n* = 20	Transition set *n* = 13
Female, *n* (%)	120 (82.2)	20 (74.1)	90 (84.9)	16 (80.0)	10 (76.9)
Age, mean (SD), years	31.9 (9.4)	33.4 (10.1)	32.1 (9.3)	31.9 (6.5)	27.7 (8.0)
Time from PAH diagnosis to selexipag initiation, medium (Q1, Q3)	36 (12, 72)	–	48 (23, 84)	5 (4, 12)	48 (31, 60)
PAH etiology, *n* (%)					
IPAH/HPAH	90 (61.6)	22 (81.5)	61 (57.5)	14 (70.0)	7 (53.8)
CHD-PAH (post-operative)	29 (19.9)	2 (7.4)	21 (19.8)	1 (5.0)	6 (46.2)
CTD-PAH	27 (18.5)	3 (11.1)	24 (22.7)	5 (25.0)	0

Continuous data are expressed as the mean ± SD or if not normally distributed as the median (Q1, Q3). IPAH, idiopathic pulmonary arterial hypertension; HPAH, heritable PAH; CHD-PAH, PAH associated with congenital heart disease; CTD-PAH, PAH associated with connective tissue diseases.

**Table 2:** Risk assessment parameters of the efficacy set at baseline and follow-up.

Variable	Baseline *n* = 146	Follow-up *n* = 146	*P*
WHO FC, *n* (%)			<0.001
I	0 (0.0)	19 (13)	
II	39 (26.7)	96 (65.8)	
III	107 (73.3)	30 (20.5)	
IV	0 (0)	1 (1.6)	
6MWD, mean ± SD, m	414 (108)	480 (420, 506)	<0.001
NT-proBNP, median (Q1, Q3), pg/mL	928 (307, 1,923)	455 (134, 1,678)	<0.001
RAA, median (Q1, Q3), cm^2^^†^	23 (17, 29)	21 (15, 33)	<0.001
RV, mean ± SD, mm^†^	38 ± 9	35 ± 10	<0.001
TAPSE, mean ± SD, mm^†^	17 ± 4	18 ± 4	<0.001
RAA < 18 cm^2^, *n* (%)	37 (25.3)	55 (37.7)	0.023
RV < 28 mm, *n* (%)	9 (6.2)	34 (23.3)	<0.001
TAPSE ≥ 18 mm, *n* (%)	56 (38.4)	93 (63.7)	<0.001
Right heart remodeling reversion, *n* (%)	5 (3.4)	31 (21.2)	<0.001
TBIL, median (Q1, Q3), mol/L^†^	12.5 (8.2, 23.4)	9.6 (11.6, 19.3)	0.003

Continuous data are expressed as the mean ± SD or if not normally distributed as the median (Q1, Q3) and compared using paired-t test or Wilcoxon matched-pairs signed rank test. Categorical data are compared using Fisher's exact test. WHO-FC, World Health Organization functional class; 6MWD, six-minute walking distance; NT-proBNP, *N*-terminal pro B-type natriuretic peptide; RAA, right atrial area; RV, right ventricle diameter; TAPSE, tricuspid annular plane systolic excursion; TBIL, total bilirubin.

† Data missing for one subject.

### Changes to the right heart in the efficacy cohort

As shown in Table 2, right heart echocardiographic parameters improved as well, with smaller RAA (23 (17, 29) vs. 21 (15, 33) cm^2^, *p* < 0.001) and RV (38 ± 9 vs. 35 ± 10 mm, *p* < 0.001) being achieved as well as improved TAPSE (17 ± 4 vs. 18 ± 4 mm, *p* < 0.001). The percentage of normal RAA increased from 25.3% to 37.7% (*p* = 0.023), normal RV increased from 6.2% to 23.3% (*p* < 0.001) and normal TAPSE increased from 56% to 93% (*p* < 0.001). Overall, only around 20% of patients achieved complete right heart remodeling reversion (31 (21.2%) vs. 5 (3.4%) patients) compared to baseline (*p* < 0.001). In light of the potential confounding effect of left-to-right shunt on right heart morphology, five patients with a small atrial septal defect (< 2 cm) were excluded from the comparative analysis presented in Table 3: one patient from the initial group and four from the sequential group.

**Table 3:** Comparison of changes from baseline to the first follow-up in risk stratification and echocardiographic parameters between initial and sequential selexipag triple combination therapy.

Parameter changes	Initial set *n* = 26^§^	Sequential set *n* = 102^§^	Early sequential set *n* = 20	*P*
6MWD, m	73 (36, 139)	41 (10, 110)	64 (26, 135)	0.021
NT-proBNP, pg/mL	−739 (−1,612, −457)	−160 (−719, −14)	−315(−1,568, −51)	0.001
WHO FC I/II, *n* (%)	18 (69.2)	56 (54.9)	15 (75)	0.003
Met 3 low-risk criteria, *n* (%)	18 (69.2)	34 (33.3)	14 (70)	0.000
RAA, cm^2^	−5 (−11, 0)	−1 (−4, 2)	−5 (−6.5)	0.013
RV, mm	−8 (−12, −3)	−3 (−6, 1)	−4 (−9, −3)	0.001
TAPSE, mm	3 (1, 6)	1 (−1, 3)	2 (−1, 7)	0.002

Continuous data are expressed as the median (lower quartile, upper quartile) and compared using Mann–Whitney test. Categorical data are compared using Fisher's exact test. IPAH, idiopathic pulmonary arterial hypertension; HPAH, heritable PAH; CHD-PAH, PAH associated with congenital heart disease; CTD-PAH, PAH associated with connective tissue diseases; 6MWD, six-minute walking distance; NT-proBNP, *N*-terminal pro B-type natriuretic peptide; RAA, right atrial area; RV, right ventricle diameter; TAPSE, tricuspid annular plane systolic excursion; WHO-FC, World Health Organization functional class.

§ Patients with a small atrial septal defect (< 2 cm) were excluded from the comparation, with one patient in the initial group and four patients in the sequential group.

The initial set demonstrated superior exercise capacity and right heart structure improvement compared with the sequential set. However, it did not surpass the early sequential set in terms of risk stratification or right heart remodeling (Table 3 and Supplementary Tables S5, S6).

### Dosage tolerance and adverse effects

In the safety cohort, 33 (21.6%) patients had selexipag withdraw. Fourteen (42.5%) patients were unwilling to continue with selexipag due to economic burden. Among these, six patients experienced deterioration in right heart function and subsequently commenced treatment with subcutaneous treprostinil. Twelve (36.4%) patients had PAH worsening and switched to subcutaneous/intravenous treprostinil, of which, 6 patients succumbed within 12 months. There are 7 (21.2%) patients stopped selexipag within 3 days due to recognized prostacyclin-related adverse events: headache (3 patients), diarrhea (2 patients), and pain in extremity and rash (1 patient each).

The majority of patients (92.8%) reported various adverse effects during selexipag titration. During selexipag maintenance, 41.2% patients reported adverse effects, of which 20 (32.8%) patients suffered from 2 or more adverse effect. The reported adverse effects during selexipag maintenance were headache (37.7%), followed by nausea/vomiting/diarrhea (27.9%), myalgia/fatigue/pain in extremity (21.3%) and jaw pain (13.1%). No other adverse effects were reported.

In the efficacy set, 47 (32.2%) patients titrated to 200–400 µg twice daily, 74 (50.7%) patients reached 600–1,000 µg twice daily, and 25 (17.1%) patients received 1,200–1,600 µg twice daily.

### Comparison between initial and sequential selexipag add-on strategy on risk status and right heart remodeling

As illustrated in Figures 2B,C, a greater proportion of patients receiving initial triple therapy achieved three low-risk index numbers (81.5% vs. 44.3%) compared to those receiving sequential selexipag add-on therapy. In other words, compared to sequential therapy, initial triple therapy had more patients improve to 3 low-risk criteria (changes of proportion, 69.2% vs. 33.3%, *p* = 0.0001) at follow-up. Additionally, there were more pronounced changes observed in RAA (−5 (−11, 0) vs. −1 (−4, 2) cm^2^, *p* = 0.013), right ventricle diameter (−8 (−12, −3) vs. −3 (−6, 1) mm, *p* = 0.001), and TAPSE (3 (1, 6) vs. 1 (−1, 3) mm, *p* = 0.002) (Table 3).

### Clinical worsening events

**Figure 3:**
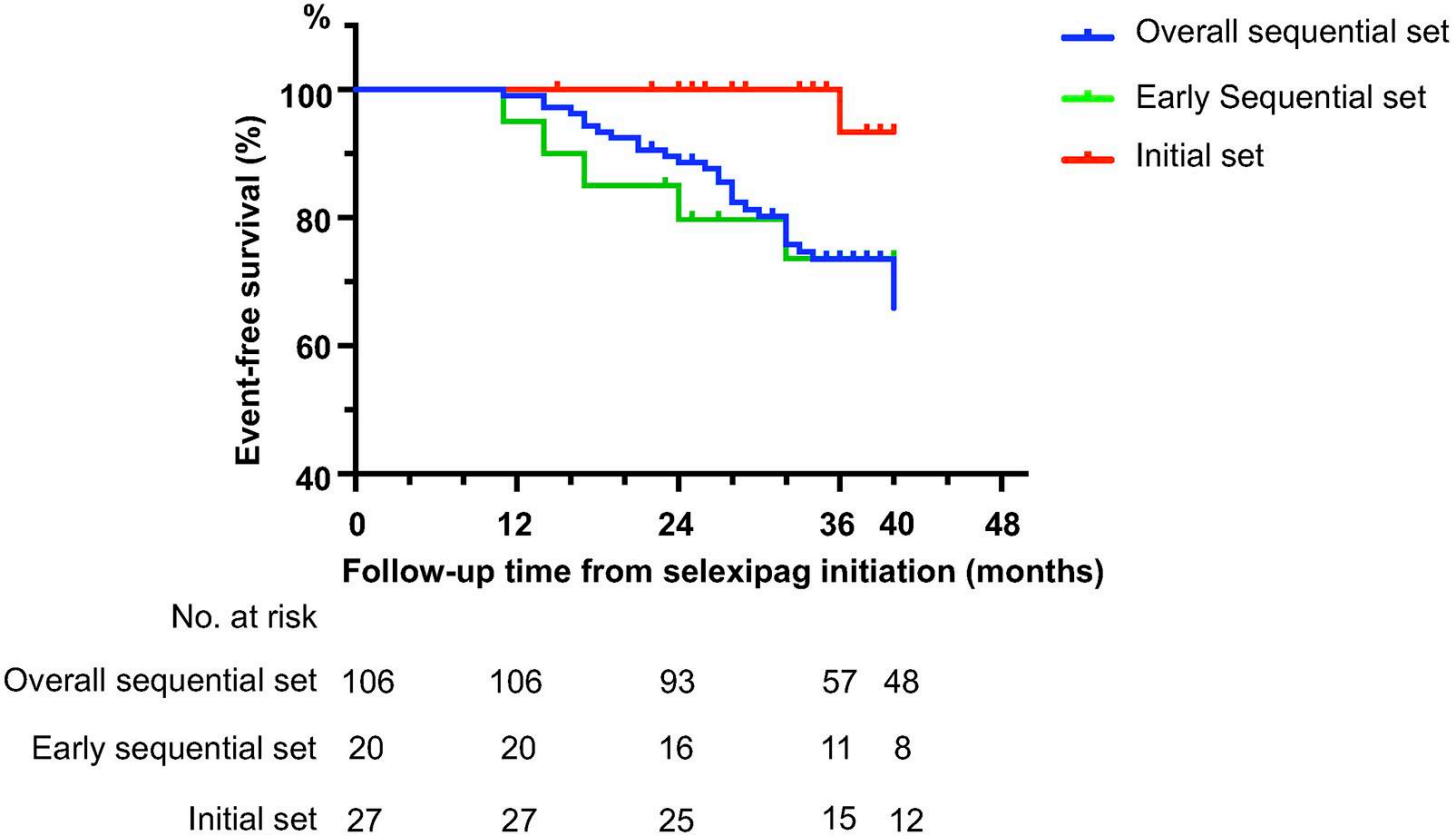
Event-free survival in selexipag-treated Chinese PAH patients. Kaplan–Meier curve for time from selexipag initiation to first clinical worsening event associated with PAH up to the cutoff date of May 31, 2025. Event-free survival of patients in the initial set, early sequential set and overall sequential set were calculated separately.

The mean follow-up period was 33 ± 10 months. During this time, 34 patients experienced a decline in right heart function, with 29 of them subsequently starting treprostinil therapy. There were 13 deaths recorded during the follow-up, with 2 patients on selexipag while the remaining 11 patients had already transitioned to treprostinil treatment. In the sequential set, 31 patients showed clinical deterioration, with 5 of them in the early sequential set. One patient in the initial cohort experienced deterioration of right heart function and switched to treprostinil. The baseline characteristics between initial and early sequential sets were comparable (Supplementary Table S1). The hazard ratio for the risk of experiencing a first disease progression event was 0.177 (*p* = 0.036, 95% CI: 0.035–0.898) for initial triple combination therapy, compared to the early sequential set (Figure 3).

## Discussion

As an oral selective IP receptor agonist, selexipag has been proven effective for PAH treatment (8, 15, 16). This study adds to existing evidence on selexipag's outcomes and safety in Chinese PAH patients. It also explores timing of initiation and effects on right heart remodeling. The *post-hoc* analysis and the emulated randomized trial, utilizing data from the US Komodo claims database demonstrated that the early addition of selexipag to an ERA plus PDE5i regimen was associated with a decreased risk of hospitalization and disease progression, particularly within the first three months (11). However, the TRITON study revealed that initial triple combination therapy comprising selexipag, macitentan, and tadalafil does not demonstrate superiority over the initial double combination of macitentan and tadalafil in reducing pulmonary vascular resistance (PVR). According to the 2022 ESC/ERS guidelines for PH, initial triple combination therapy involving selexipag is not recommended due to the unfavorable outcome of the primary endpoint (4). Nonetheless, the TRITON study also indicated that initial triple combination therapy with selexipag may potentially reduce the risk of disease progression (17). Further *post hoc* analyses that pooled newly diagnosed (≤6 months) PAH patients from the GRIPHON and TRITON studies also suggested that selexipag delayed disease progression (18).

In the present study, we compared the efficacy of an upfront triple combination therapy with selexipag to a sequential combination. The results indicate a potential advantage of the upfront add-on strategy. However, the sequential group consisted of numerous patients with a prolonged disease course, suggesting a delay in treatment and an elevated risk of disease progression. Consequently, we compared the initial group with an early sequential group, which included patients who received an initial double combination therapy and commenced selexipag as an add-on within 12 months following a PAH diagnosis. Consistent with the findings of the TRITON study, the initial strategy did not demonstrate any advantage in terms of exercise capacity or risk stratification when compared to the early sequential strategy within the first six months during follow-up. Two major differences distinguish the study populations of our analysis from those of the TRITON trial. Firstly, the patient cohort in the TRITON study was, on average, 20 years older than those in the current study, and it included a higher proportion of cases with CTD-PAH. The analysis of the EXPOSURE study revealed that the rate of patients free from hospitalization at 1 year was only 63% among CTD-PAH patients initiating selexipag (19). These differences in age and etiology may explain the observed variation (Supplementary Table S1) (20) which is lower than the 76% observed in the overall population (21). Secondly, none of the patients in the initial set exhibit any cardiovascular comorbidities, which is unusual in EU and US studies. This is possibly because the patients involved were predominantly young as has been observed (22). Although the TRITON study does not provide detailed information regarding comorbidities, it is plausible that similar conditions apply, given that exclusion criteria included the presence of three or more risk factors for heart failure with preserved ejection fraction (BMI greater than 30 kg/m^2^, diabetes mellitus of any type, essential hypertension, and coronary artery disease) at the time of screening. In this retrospective study, the initial strategy was associated with a trend toward delayed disease progression; however, this comparison was not prespecified and involved small subgroup sizes, it is considered exploratory; therefore, the observed effect estimate must be interpreted with caution and cannot support definitive conclusions regarding treatment benefit. Independent validation in adequately powered, prospective trials is required.

Echocardiographic imaging is an essential component of current multimodality assessment of right heart structure and function. Echocardiographic parameters such as a decrease in RV end-diastolic area (RVEDA), RAA, and left ventricular systolic eccentricity index (LV-EIs) after 1 year of treatment are independent predictors of long-term survival in IPAH (23). The Delphi study agreed that improving or normalizing right heart imaging may help to better evaluate patients' progress earlier than clinical assessments (24). In our previous exploratory study with a small sample size, although the triple combination with selexipag improved risk status, the reduction in RAA did not reach statistical significance (9). In the present study, which features a larger sample size, we have demonstrated that selexipag treatment improved both the RAA and TAPSE as measured by echocardiogram, both of which are predictors of PAH survival. However, the proportion of patients who achieved reversal in both RAA and TAPSE remained low.

A reduction in PVR was identified as the sole independent determinant of right heart reverse remodeling (23). In the TRITON study, initial triple combination therapy with selexipag demonstrated a reduction in PVR comparable to that achieved with the initial double combination therapy. However, initial combination therapy from the current study exhibited superior event-free survival compared to the sequential combination. The relationship between PVR reduction and survival in patients with PAH is complex and not linear. The right ventricle's capacity to adapt to increased afterload and maintain adequate cardiac output may be more critical for patient survival than the absolute PVR value (13). It seems that in addition to limited PVR reduction in a relatively short time frame, selexipag also decelerated the progression of pulmonary vascular remodeling over the long term.

An additional noteworthy observation is that within the early sequential group, all patients initially received a double combination therapy. However, after a mean treatment duration of 5 months, just prior to the initiation of selexipag, only 15% of patients met all three low-risk criteria. Several real-world studies support the notion that initial double combination therapy may be inadequate for achieving the primary objective of maintaining a low-risk status. For instance, only 43% of patients in an Italian cohort achieved low-risk status with initial double combination (5). Furthermore, data from the EXPOSURE study indicated that among those who commenced initial double therapy, 17% escalated to triple therapy within 7 months (25). On the other hand, there is cumulative evidence to support initial triple combination including the prostacyclin pathway (6, 7).

## Study limitations

This study has several limitations. First, its retrospective design precludes longitudinal assessment of right heart remodeling, thereby limiting the strength of causal inference regarding the impact of initial triple combination therapy. Second, the sample size is limited due to constraints in both overall cohort size and the number of endpoint events. Consequently, the low event count—only six events in total across the initial and early sequential treatment groups—precludes reliable estimation using multivariable Cox regression. According to the widely accepted Events Per Variable (EPV) rule of thumb, a minimum of 10 endpoint events is required per covariate adjusted for in the model; therefore, inclusion of even a single confounder would violate this threshold. Third, baseline right heart catheterization was not mandated, resulting in incomplete acquisition of invasive hemodynamic parameters (e.g., mean pulmonary arterial pressure, pulmonary vascular resistance, cardiac index); consequently, these metrics could not be incorporated into comparative or multivariable analyses. Notably, although PAH is a progressive disease and even low-risk patients remain vulnerable to clinical deterioration, we adopted an early triple combination strategy incorporating selexipag based on pathophysiological rationale and emerging clinical experience. However, the current evidence, derived from a relatively small cohort, remains insufficient to robustly support routine use of this approach in low-risk PAH patients. Given the high cost of selexipag and uncertainties regarding long-term benefit–risk balance (26), prospective studies with larger sample sizes, standardized serial assessments of right heart structure and function (e.g., via echocardiography and cardiac MRI), and pre-specified clinical endpoints are needed to rigorously evaluate the effects of early triple therapy on right heart remodeling and hard outcomes. Such studies should also aim to identify predictive biomarkers or clinical phenotypes that enrich for low-risk patients most likely to derive sustained benefit thereby advancing precision medicine in PAH management.

## Data Availability

The raw data supporting the conclusions of this article will be made available by the authors, without undue reservation.
