# Supplementary material for: Upfront triple combination therapy with selexipag: insights from a real world cohort in Chinese patients with pulmonary arterial hypertension
Source: Front Cardiovasc Med. 2026 May 21;13:1745171. doi: 10.3389/fcvm.2026.1745171 (PMC13233463; doi:10.3389/fcvm.2026.1745171)
Supplement: Supplementary file 1 [file Table1.docx]

**Supplemental table 1. Comparative risk assessment between baseline and follow-up conducted 13-19 weeks post-selexipag initiation**

|  | **Baseline**  **n=28** | **Follow-up**  **n=28** | ***P*** |
| --- | --- | --- | --- |
| **WHO FC** I/II**, n (%)** | 8 (28.6%) | 21 (75.0%) | <0.001 |
| **6MWD, mean (SD), m** | 415.43 ± 96.98 | 454.68 ± 124.85 | 0.109 |
| **NT-proBNP, median (Q1, Q3), pg/mL** | 910.50 (234.75, 2207.5) | 201.00 (70.63, 639.50) | <0.001 |
| **Number of low risk indices** |  |  | 0.024 |
| 0 | 13 (46.4%) | 6 (21.4%) |  |
| 1 | 7 (25.0%) | 3 (10.7%) |  |
| 2 | 2 (7.1%) | 2 (7.1%) |  |
| 3 | 6 (21.4%) | 17 (60.7%) |  |
| 0 | 13 (46.4%) | 6 (21.4%) |  |

Continuous data are expressed as the mean (SD) or if not normally distributed as the median (Q1, Q3) and compared using paired-t test or Wilcoxon matched-pairs signed rank test. Categorical data are compared using Fisher's exact test. * *P* < 0.05 between two groups. WHO-FC, World Health Organization functional class; 6MWD, six-minute walking distance; NT-proBNP, *N*-terminal pro B-type natriuretic peptide.
