# Supplementary material for: Upfront triple combination therapy with selexipag: insights from a real world cohort in Chinese patients with pulmonary arterial hypertension
Source: Front Cardiovasc Med. 2026 May 21;13:1745171. doi: 10.3389/fcvm.2026.1745171 (PMC13233463; doi:10.3389/fcvm.2026.1745171)
Supplement: Supplementary file 2 [file Table2.docx]

**Supplemental table 2. Comparative risk assessment between baseline and follow-up conducted 20-28 weeks post-selexipag initiation**

|  | **Baseline**  **n = 42** | **Follow-up**  **n = 42** | ***P*** |
| --- | --- | --- | --- |
| **WHO FC** I/II**, n (%)** | 7 (16.7) | 28 (66.7) | <0.001 |
| **6MWD, mean (SD), m** | 383.9 ± 114.2 | 447.1 ± 115.4 | 0.001 |
| **NT-proBNP, median (Q1, Q3), pg/mL** | 1089.0 (422.7, 2431.5) | 320.1 (88.4, 1546.5) | <0.001 |
| **Number of low risk indices, n (%)** |  |  | <0.001 |
| 0 | 26 (61.9) | 9 (21.4) |  |
| 1 | 8 (19.0) | 6 (14.3) |  |
| 2 | 7 (16.7) | 10 (23.8) |  |
| 3 | 1 (2.4) | 17 (40.5) |  |

Continuous data are expressed as the mean (SD) or if not normally distributed as the median (Q1, Q3) and compared using paired-t test or Wilcoxon matched-pairs signed rank test. Categorical data are compared using Fisher's exact test. * *P* < 0.05 between two groups. WHO-FC, World Health Organization functional class; 6MWD, six-minute walking distance; NT-proBNP, *N*-terminal pro B-type natriuretic peptide.
