# Supplementary material for: Upfront triple combination therapy with selexipag: insights from a real world cohort in Chinese patients with pulmonary arterial hypertension
Source: Front Cardiovasc Med. 2026 May 21;13:1745171. doi: 10.3389/fcvm.2026.1745171 (PMC13233463; doi:10.3389/fcvm.2026.1745171)
Supplement: Supplementary file 3 [file Table3.docx]

**Supplemental table 3. Comparative risk assessment between baseline and follow-up conducted ≥ 29 weeks post-selexipag initiation**

|  | **Baseline**  **n = 76** | **Follow-up**  **n = 76** | ***P*** |
| --- | --- | --- | --- |
| **WHO FC** I/II**, n (%)** | 24 (31.6) | 66 (86.8) | <0.001 |
| **6MWD, mean (SD), m** | 429.9 ± 106.2 | 491.8 ± 74.4 | <0.001 |
| **NT-proBNP, median (Q1, Q3), pg/mL** | 822.0 (191.3, 1581.0) | 196.5 (94.6, 787.5) | <0.001 |
| **Number of low risk indices, n (%)** |  |  | <0.001 |
| 0 | 32 (42.1) | 8 (10.5) |  |
| 1 | 17 (22.4) | 5 (6.6) |  |
| 2 | 16 (21.1) | 21 (27.6) |  |
| 3 | 11 (14.5) | 42 (55.3) |  |

Continuous data are expressed as the mean (SD) or if not normally distributed as the median (Q1, Q3) and compared using paired-t test or Wilcoxon matched-pairs signed rank test. Categorical data are compared using Fisher's exact test. * *P* < 0.05 between two groups. WHO-FC, World Health Organization functional class; 6MWD, six-minute walking distance; NT-proBNP, *N*-terminal pro B-type natriuretic peptide.
