# Supplementary material for: Upfront triple combination therapy with selexipag: insights from a real world cohort in Chinese patients with pulmonary arterial hypertension
Source: Front Cardiovasc Med. 2026 May 21;13:1745171. doi: 10.3389/fcvm.2026.1745171 (PMC13233463; doi:10.3389/fcvm.2026.1745171)
Supplement: Supplementary file 4 [file Table4.docx]

**Supplemental table 4. Risk assessment parameters of the transition set at baseline and follow-up.**

|  | **Baseline** | **Follow-up** | ***P*** |
| --- | --- | --- | --- |
| **WHO FC, n (%)** |  |  | 0.48 |
| I | 0 (0.0) | 1(7.7) |  |
| II | 7 (53.8) | 8 (61.5) |  |
| III | 6 (46.2) | 4 (30.8) |  |
| IV | 0 (0.0) | 0 (0.0) |  |
| **6MWD, mean ±** **SD, m** | 426 (77) | 433（119） | 0.856 |
| **NT-proBNP, median (Q1, Q3), pg/mL** | 900 (260, 1736) | 273 (88, 924) | 0.055 |
| **RAA, median (Q1, Q3), cm^2^** | 28 (21, 35) | 24 (17, 39) | 0.102 |
| **RV, mean ±** **SD, mm** | 40 (9) | 38 (10) | 0.224 |
| **TAPSE, mean ±** **SD, mm** | 18(3) | 19 (3) | 0.391 |

Continuous data are expressed as the mean (SD) or if not normally distributed as the median (Q1, Q3) and compared using paired-t test or Wilcoxon matched-pairs signed rank test. Categorical data are compared using Fisher's exact test. WHO-FC, World Health Organization functional class; 6MWD, six-minute walking distance; NT-proBNP, *N*-terminal pro B-type natriuretic peptide; RAA, right atrial area; RV, right ventricle diameter; TAPSE, tricuspid annular plane systolic excursion.
