# Supplementary material for: Upfront triple combination therapy with selexipag: insights from a real world cohort in Chinese patients with pulmonary arterial hypertension
Source: Front Cardiovasc Med. 2026 May 21;13:1745171. doi: 10.3389/fcvm.2026.1745171 (PMC13233463; doi:10.3389/fcvm.2026.1745171)
Supplement: Supplementary file 5 [file Table5.docx]

**Supplemental table 5. Baseline characteristics comparison between current study and TRITON study**

|  | **Initial set**  **n=26** | **Early sequential set**  **n=20** | **Initial triple set from TRITON study**  **n=123** |
| --- | --- | --- | --- |
| **Female, n (%)** | 20 (74.1) | 16 (80.0) | 93 (75.6) |
| **Age, mean (SD), years** | 33.4 (10.1) | 31.9 (6.5) | 52.2 (13.5) |
| **Time from PAH diagnosis to selexipag initiation, medium (Q1, Q3)** | -- | 5 (4, 12) | -- |
| **PAH etiology, n (%)** |  |  |  |
| IPAH/HPAH | 22 (81.5) | 14 (70.0) | 61 (51.4) |
| CHD-PAH (post-operative) | 2 (7.4) | 1 (5.0) | 1 (0.8) |
| CTD-PAH | 3 (11.1) | 5 (25.0) | 43 (35) |
| Drug/toxin induced &HIV-PAH | 0 | 0 | 17 (13.8) |
| **Low-risk criteria, n (%)** |  |  |  |
| 3 | 4 (14.8) | 3 (15.0) |  |
| 2 | 5 (18.6) | 3 (15.0) |  |
| 1 | 6 (22.2) | 5 (25.0) |  |
| 0 | 12 (44.4) | 9 (45.0) |  |

IPAH, idiopathic pulmonary arterial hypertension; HPAH, heritable PAH; CHD-PAH associated with congenital heart disease; CTD-PAH, PAH associated with connective tissue diseases; HIV, human immunodeficiency virus.
