# Supplementary material for: Upfront triple combination therapy with selexipag: insights from a real world cohort in Chinese patients with pulmonary arterial hypertension
Source: Front Cardiovasc Med. 2026 May 21;13:1745171. doi: 10.3389/fcvm.2026.1745171 (PMC13233463; doi:10.3389/fcvm.2026.1745171)
Supplement: Supplementary file 6 [file Table6.docx]

**Supplemental Table 6. Comparison from baseline to the first follow-up in risk stratification and echocardiographic parameters between initial and sequential selexipag triple combination therapy.**

| Parameter Changes | Initial set  n = 26 ^§^ | | |  | Sequential set  n = 102 ^§^ | | |  | Early sequential set  n = 20 | | |
| --- | --- | --- | --- | --- | --- | --- | --- | --- | --- | --- | --- |
|  | Baseline | Follow-up | *P* value |  | Baseline | Follow-up | *P* value |  | Baseline | Follow-up | *P* value |
| 6MWD, m | 419 ± 123 | 522 ± 61 | 0.0004 |  | 432 (349, 470) | 480 (420, 520) | < 0.0001 |  | 436 (393, 493) | 518 (483, 556) | 0.003 |
| NT-proBNP, pg/ml | 876 (576, 1898) | 102 (56, 180) | < 0.0001 |  | 946 (275, 2011) | 257 (97, 1416) | 0.0014 |  | 468 (112, 1612) | 68 (41, 209) | < 0.0001 |
| WHO FC I/II | 8 (30.8) | 26 (100) | < 0.0001 |  | 20 (19.6) | 76 (74.5) | < 0.0001 |  | 5 (25) | 20 (100) | < 0.0001 |
| Met 3 low-risk criteria, n (%) | 4 (15.4) | 22 (84.6) | < 0.0001 |  | 11 (10.8) | 45 (44) | < 0.0001 |  | 3 (15) | 17 (85) | < 0.0001 |
| RAA, cm^2^ | 20 (15, 27) | 16 (13, 20) | 0.013 |  | 23 (18, 29) | 20 (16, 28) | 0.0536 |  | 22 ± 7 | 17 ± 4 | 0.0099 |
| RV, mm | 34 (32, 41) | 28 (25, 34) | 0.0002 |  | 37 (32, 44) | 33 (29, 41) | 0.0061 |  | 36 (29, 43) | 29 (26, 32) | 0.0022 |
| TAPSE, mm | 16 (14, 17) | 19 (17, 21) | < 0.0001 |  | 17 ± 4 | 18 ± 4 | 0.0216 |  | 17 ± 4 | 20 ± 3 | 0.017 |

Continuous data are expressed as the median (lower quartile, upper quartile) and compared using Mann-Whitney test. Categorical data are compared using Fisher’s exact test. * *P* < 0.05 between initial vs sequential sets. ^#^ *P* < 0.05 between initial vs early sequential sets. ^§^ Patients with a small atrial septal defect (< 2 cm) were excluded from the comparation, with one patient in the initial group and four patients in the sequential group. IPAH, idiopathic pulmonary arterial hypertension; HPAH, heritable PAH; CHD-PAH, PAH associated with congenital heart disease; CTD-PAH, PAH associated with connective tissue diseases; 6MWD, six-minute walking distance; NT-proBNP, *N*-terminal pro B-type natriuretic peptide; RAA, right atrial area; RV, right ventricle diameter; TAPSE, tricuspid annular plane systolic excursion; WHO-FC, World Health Organization functional class.
